# North polar trough formation due to in-situ erosion as a source of young ice in mid-latitudinal mantles on Mars

**DOI:** 10.1038/s41598-021-83329-3

**Published:** 2021-03-25

**Authors:** J. Alexis P. Rodriguez, Kenneth L. Tanaka, Ali M. Bramson, Gregory J. Leonard, Victor R. Baker, Mario Zarroca

**Affiliations:** 1 Planetary Science Institute, 1700 East Fort Lowell Road, Suite 106, Tucson, AZ 85719-2395 USA; 2 Department of Earth, Atmospheric, and Planetary Sciences, Purdue University, 550 Stadium Mall Dr, West Lafayette, IN 47907 USA; 3 Department of Planetary Sciences, Lunar and Planetary Laboratory, University of Arizona, Tucson, AZ 85721 USA; 4 Department of Hydrology and Atmospheric Sciences, University of Arizona, Tucson, AZ 85721 USA; 5 External Geodynamics and Hydrogeology Group, Department of Geology, Autonomous University of Barcelona, 08193 Bellaterra, Barcelona Spain

**Keywords:** Planetary science, Geomorphology

## Abstract

The clockwise spiral of troughs marking the Martian north polar plateau forms one of the planet’s youngest megastructures. One popular hypothesis posits that the spiral pattern resulted as troughs underwent poleward migration. Here, we show that the troughs are extensively segmented into enclosed depressions (or cells). Many cell interiors display concentric layers that connect pole- and equator-facing slopes, demonstrating in-situ trough erosion. The segmentation patterns indicate a history of gradual trough growth transversely to katabatic wind directions, whereby increases in trough intersections generated their spiral arrangement. The erosional event recorded in the truncated strata and trough segmentation may have supplied up to ~25% of the volume of the mid-latitude icy mantles. Topographically subtle undulations transition into troughs and have distributions that mimic and extend the troughs’ spiraling pattern, indicating that they probably represent buried trough sections. The retention of the spiral pattern in surface and subsurface troughs is consistent with the megastructure’s stabilization before its partial burial. A previously suggested warm paleoclimatic spike indicates that the erosion could have occurred as recently as ~50 Ka. Hence, if the removed ice was redeposited to form the mid-latitude mantles, they could provide a valuable source of near-surface, clean ice for future human exploration.

## Introduction

**Figure 1:**
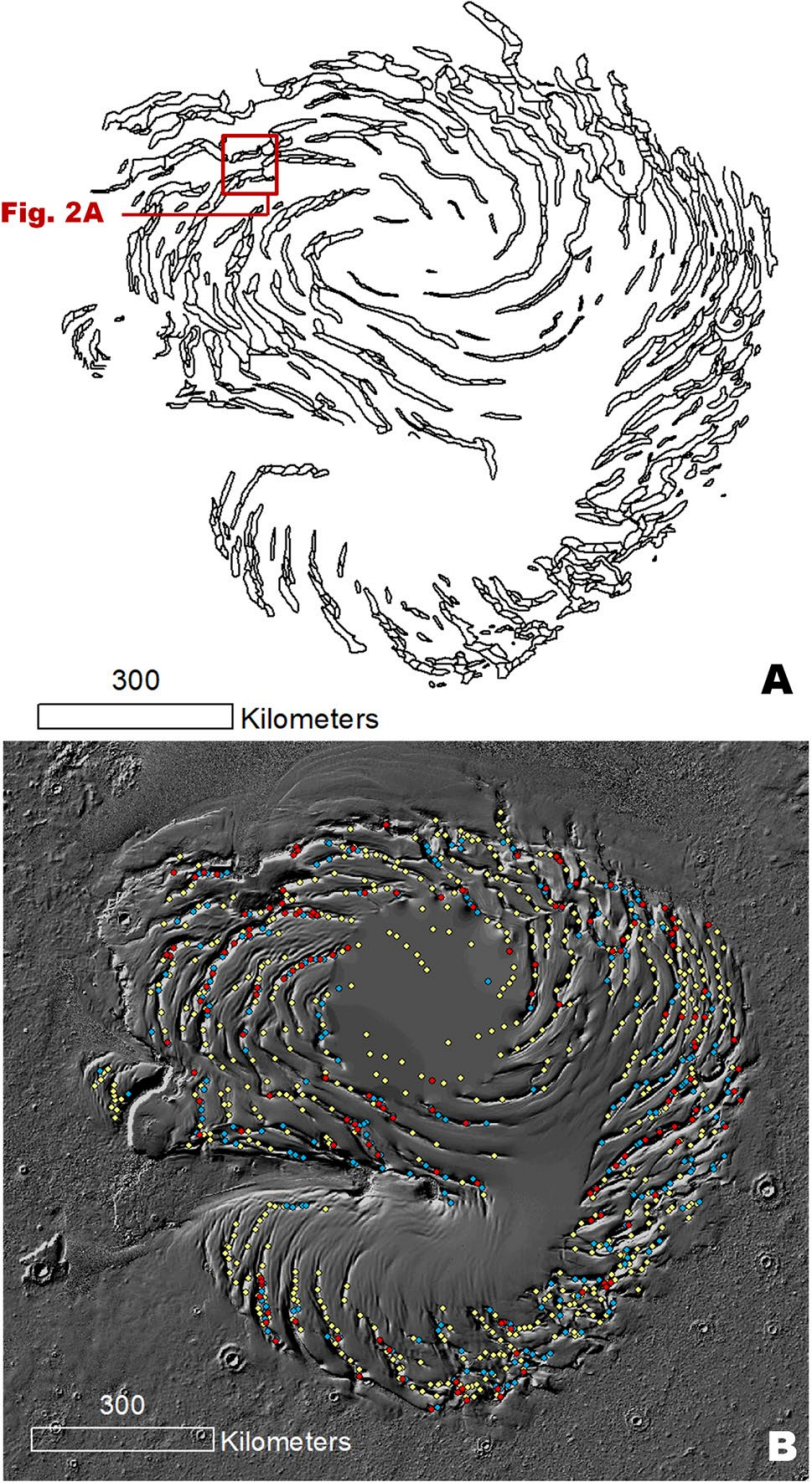
(**A**) Map of the NPLD troughs, including the outlines of their widespread constituent enclosed depressions (cells). The location of Fig. 2A is indicated. (**B**) Map showing a statistically significant population (950) of local image observations within the NPLD troughs, which demonstrates the presence of widespread trough interior surfaces produced by in-situ truncation. The observations include cell floor depressions (red dots) and mounds (blue dots) with concentrically layered exposures (including both equator- and pole-facing layers) and cells that only have equator-facing surface exposures (yellow dots). The base map is a MOLA shaded relief, illumination from the upper left (256 pixels/degree, credit: MOLA Science Team, MSS, JPL, NASA). We produced this figure using Esri’s ArcGIS 10.3 (http://www.esri.com/software/arcgis).

Planum Boreum (PB), the Martian north polar plateau, consists of a water–ice cap that features a network of troughs arranged in a clockwise spiral pattern^1,2^ (Fig. 1A). The history of PB possibly spans as much as ~ 3 billion years^1^. Its stratigraphy includes an ancient, basal sequence of poorly preserved layers overlain by local sets of buried, frozen dunes and the much younger, well-preserved, water–ice-rich north polar layered deposits (NPLD)^1^.

The NPLD consist of hundreds to thousands of layers formed by episodic ice accumulation due to cyclic climatic fluctuations with various periodicities on the order of tens and hundreds of thousands to millions of years^3–9^. A potential solar cycle of only ~ 1.5 Ka could have also modulated the depositional episodicity^7^. These polar materials consist of two distinct units^1,7^: upper and lower layered deposits (ULD and LLD, respectively) (Fig. S1). The ULD is the youngest, uppermost part of the NPLD. It consists of several high-albedo layers forming a sequence tens-of-meters thick and displays a mean crater age of 8.7 ± 6.2 Ka^1,7^. This unit covers most of the plateau’s inter-trough regions and exhibits widespread, topographically subtle (a few tens of meters deep) linear depressions (undulations, (Fig. S2)^10^). Unlike the troughs, the undulations have surfaces that lack layered exposures^10^. On the other hand, the LLD consist of moderately high-albedo layers locally resolvable down to decimeter and meter thicknesses; possibly numbering in the hundreds to thousands in the thickest sequences that reach several hundred meters of relief^1,7,10,11^. Superposed over both the ULD and LLD is the youngest unit—a tens-of-cm-thick residual ice cap and local deposits of dark sedimentary material^1,2^.

Detailed mapping of the polar plateau’s stratigraphy combined with crater count statistics suggest that the NPLD could have started to accumulate during the Middle Amazonian (~ 1 Ga)^1^ and that the trough surfaces might be at least ~ 3.6 ± 2.5 Ma^7^. This hypothesis implies that the NPLD-forming ice was stable over enormous geologic timescales, as appears to be regionally the case for the ice that forms PB’s basal unit^1^ and its surrounding zones of the Vastitas Borealis Formation (VBF), an ice-rich deposit that covers most of the planet’s northern plains^12^.

However, there is a standing disagreement between the NPLD ages derived from geologic investigations^1,2,7^ and those inferred from paleoclimatic simulations^3,5^. The latter view suggests NPLD accumulation may have started ~ 4 Ma when a decrease in Mars’ mean obliquity to ~ 25° destabilized the mid-latitude ice reservoirs, resulting in climatic conditions that facilitated water vapor transport and widespread deposition to the north pole^3,5^. In accordance with this geologic context, an estimate is that the upper few hundred meters in the NPLD, including the materials exposed within the troughs, could have formed during the last ~ 370 Ka^13.^ This is an order of magnitude younger than the ~ 3.6 ± 2.5 Ma age determined from crater counts^7^. Thus, NPLD accumulation times of millions to hundreds of millions of years all seem plausible, and previous models of NPLD accumulation all make assumptions about processes for which much uncertainty exists.

Surface geologic investigations of Viking Orbiter data^14,15^ and the later analysis of subsurface radar reflectors obtained by the Shallow Radar (SHARAD) sounder onboard the Mars Reconnaissance Orbiter^15–19^ suggest the troughs initiated about halfway through the NPLD accumulation and that they migrated poleward as further accumulation of the NPLD ensued. The hypothesized trough migration is generally explained by water–ice transfer from the troughs’ warmer equatorward-facing slopes to their colder poleward-facing slopes. The processes involved include solar insolation-driven sublimation of equatorward-facing ice, downslope transport of water vapor via katabatic winds, ice-vapor condensation onto the cold, poleward-facing slopes, and perhaps mechanical erosion and sedimentation of ice particles^14,16,17^. Simulations of the sublimation from the trough walls, combined with proposed NPLD accumulation rates recreate the trough-migration paths inferred to exist in the subsurface from radar-sounding data^20^. The trough migration process has also been invoked to explain the development of their spiral pattern as poleward migration rearranged systems of initially disorganized troughs^21^.

In this article, however, we show geologic evidence indicating that in-situ erosion led to the formation of the currently exposed troughs and undulations and that their current spiral arrangement resulted from their progressive growth and spatial integration.

## Methods

We used Esri’s ArcGIS software as the primary mapping and analytical tool for the spatial, topographic, and stratigraphic characterizations of troughs and undulations across the entire north polar plateau, Planum Boreum. Because this morphologic mapping required high-resolution images registered to an accurate digital elevation model (DEM), we used (~ 6 m/pixel) Mars Reconnaissance Orbiter Context Camera (CTX) and (~ 18 m/pixel) Thermal Emission Imaging System (THEMIS) VIS (visible light) image mosaics georeferenced to a Mars Orbiter Laser Altimeter (MOLA) DEM (~ 115 m/pixel with a one-meter vertical accuracy). We mapped the troughs’ inner segmentation into cells by topographically tracing the upper margins of the interior enclosed depressions that constitute the cells (Fig. S3). To perform the trough volume calculations, we first defined polygons around each mapped trough and extracted elevation points along their rims to produce a triangulated irregular network (TIN), representing interpolated surfaces spanning across the troughs. From this we produced a gridded reconstructed (i.e., pre-erosional) capping surface across Planum Boreum. Finally, we subtracted the troughs’ current topography from the modeled capping surface to estimate the approximate erosional volumes consequent of their excavation.

## Results

### Evidence of widespread trough interior in-situ erosion

In plan view, most NPLD troughs have simple elongate, quasi-elliptical outlines exhibiting topographic segmentation (Fig. S3). Each segment (hereon, referred to as a cell) consists of an enclosed depression outlined by equator and pole-facing slopes (letters A–E in Fig. S3), which locally flank trough interior ridges (numbers 1–3 in Fig. S3).

**Figure 2:**
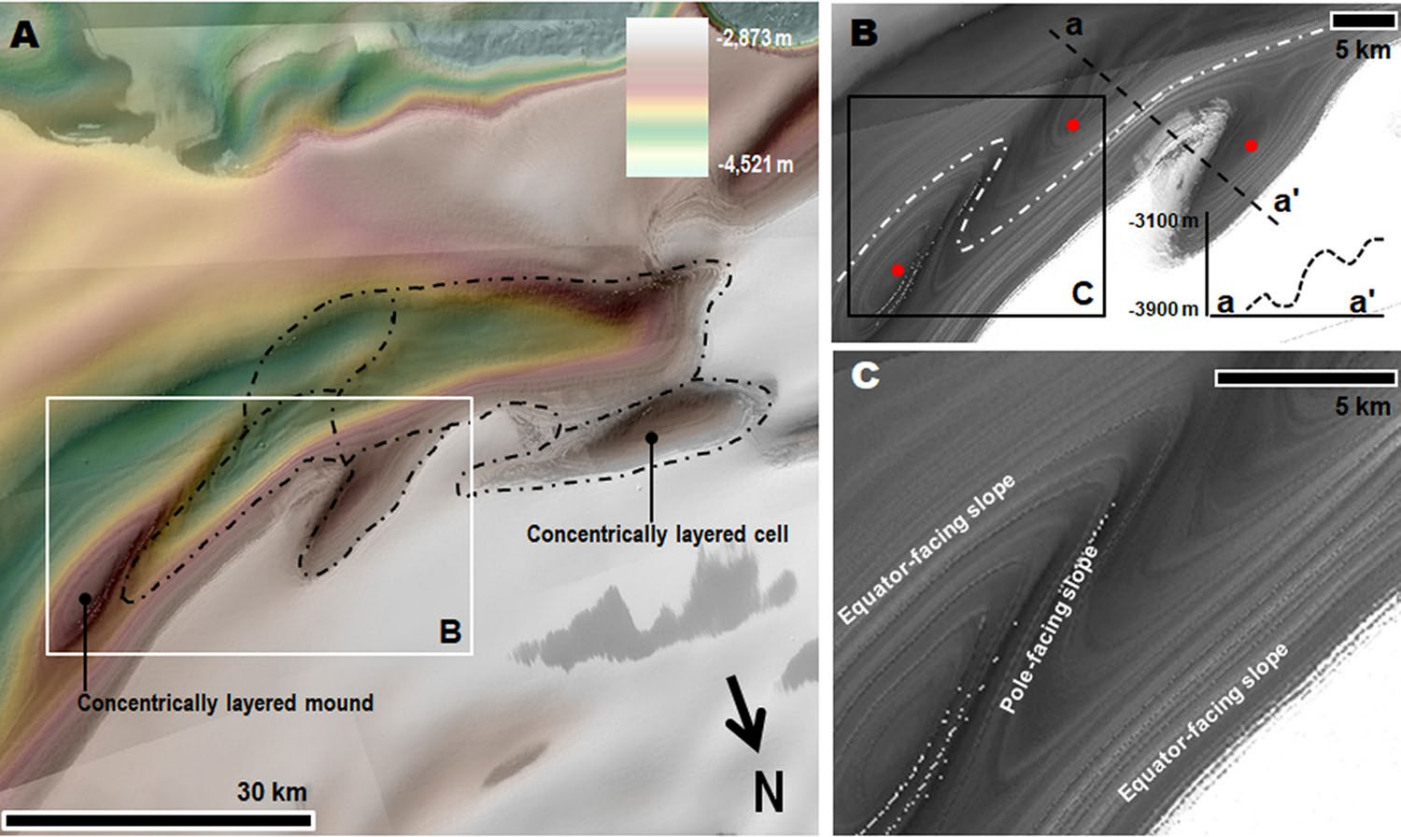
(**A**) Close-up view centered at 85° 10' N, 110° 59' W on a PB trough section. The dashed black lines trace the outlines of several individual cells. See Fig. 1A for context and location. The image is a color MOLA digital elevation model (512 pixels/degree, credit: MOLA Science Team, MSS, JPL, NASA) draped over part of a CTX mosaic (6 m/pixel, credit: NASA/JPL. The license terms can be found at pds-imaging.jpl.nasa.gov/portal/mro_mission.html). (**B**) Close-up view on panel (**A**). The dashed white line identifies a group of layers bounding two cells. The layers have equator and pole-facing exposures. The elevation profile (extracted from the MOLA DEM) shows that the trough’s total relief (~ 800 m) is bounded by these layers (red dots in panels A and B identify outcrops). (**C**) Close-up view on panel (**B**). View shows examples of layers along the margins of a cell partly nested between a layered mound and the trough’s equator-facing slope, which clearly demonstrate a truncation origin. The panels are parts of a CTX mosaic (6 m/pixel, credit: NASA/JPL. The license terms can be found at pds-imaging.jpl.nasa.gov/portal/mro_mission.html). We produced this figure using Esri’s ArcGIS 10.3 (http://www.esri.com/software/arcgis).

**Figure 3:**
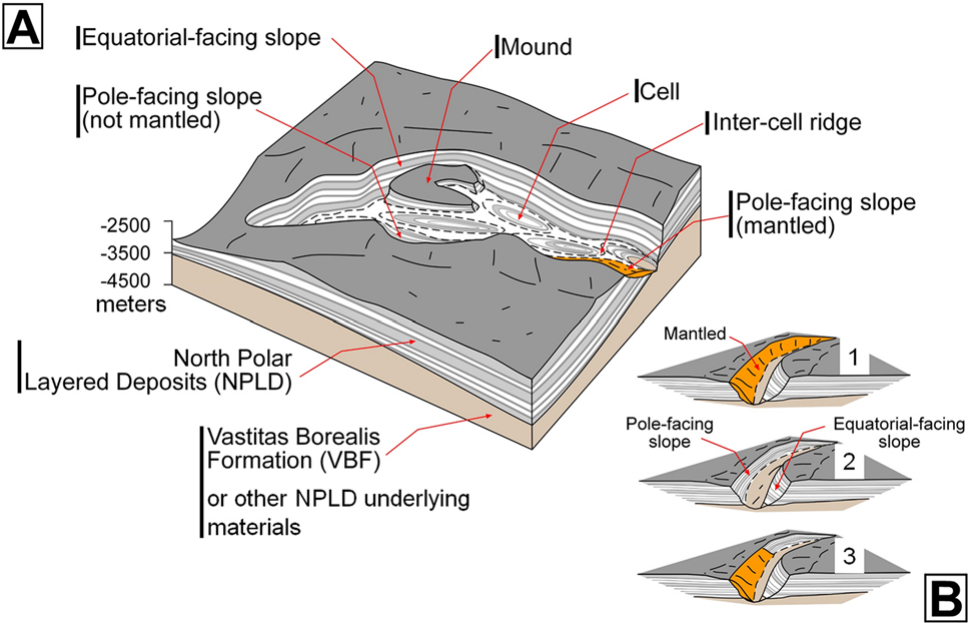
(**A**) Sketch showing a perspective view of the NPLD stratigraphy, which characterizes widespread sections of the NPLD troughs. Key morphologic features include the presence of elongate enclosed depressions (cells), inter-cell ridges, and interior mounds. The mounds and the cell walls exhibit concentrically layered exposures, indicating that the troughs’ volumes are largely due to in-situ excavation (see Fig. 2 and S4 for examples of relevant optical and topographic observations). Also indicated is the location of a pole-facing slope section where young mantles cover the truncated layered outcrops (see Figs. 4, S8, S9 for examples of relevant optical observations). (**B**) View of typical trough exposures based on the complete presence or absence of pole-facing layers (1 and 2) and troughs showing partial mantle obscuration of the underlying truncated strata (3).

**Figure 4:**
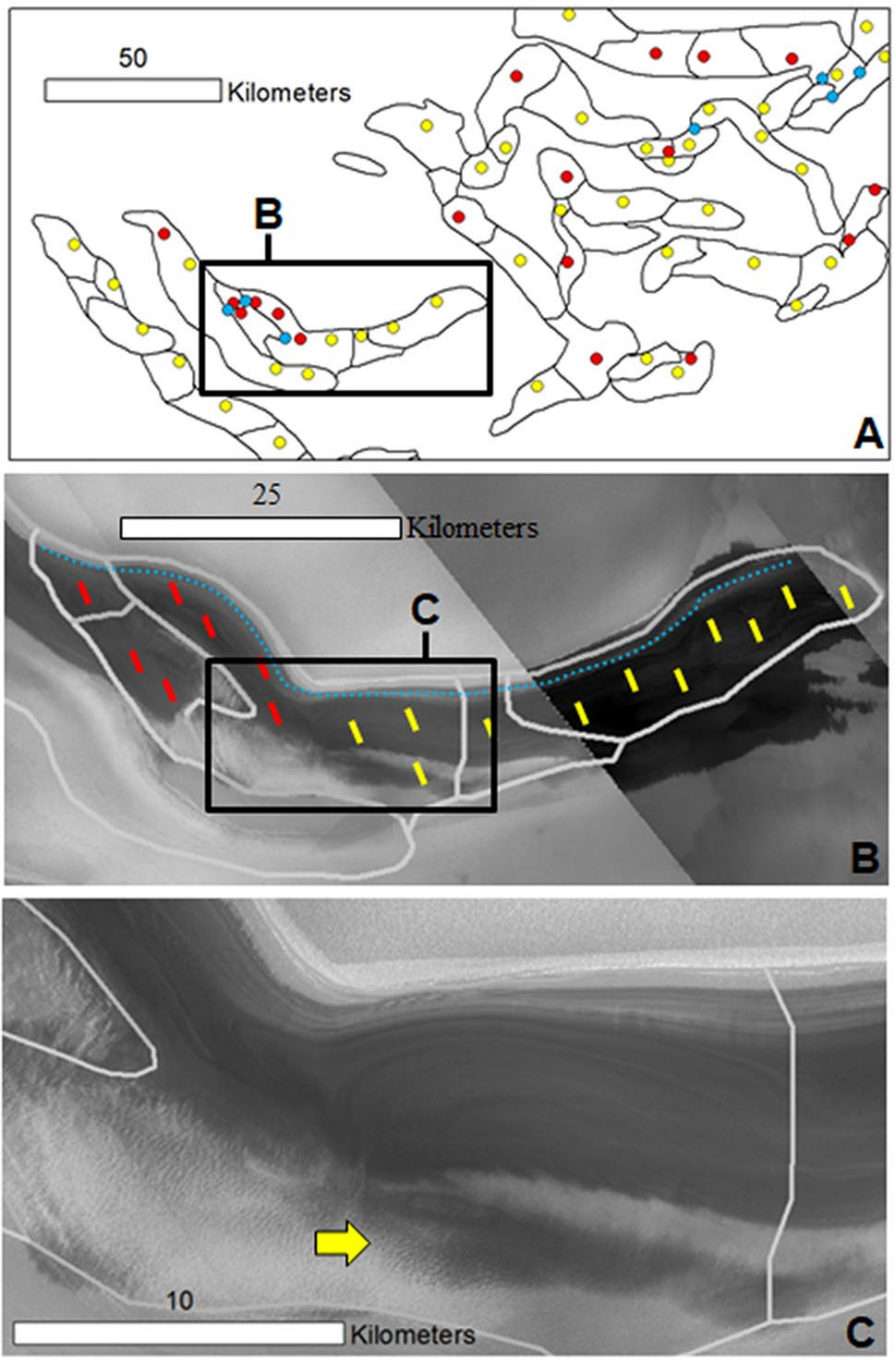
(**A**) View showing a sample of our mapping of the spiral troughs centered at 80°18' N 19°43' E. The black lines outline the cells. The red dots and blue dots, respectively, identify cell floors and mounds with both equator- and pole-facing strata. The yellow dots identify cells with only equator-facing strata exposures. (**B**) Close-up view on panel (**A**) showing a trough that includes some cells with interior outcrops that exhibit equator- and pole-facing layers (area marked by red lines) and other cells that show only equator-facing layers (area marked by yellow lines). These areas share some of the uppermost equator-facing slope layers (blue dotted line). (**C**) A close-up view on the trough in panel (**B**) shows a floor zone with equator- and pole-facing layers adjoining an area where mantles cover the pole-facing layers (yellow arrow). Panels (**B**) and (**C**) are parts of a CTX mosaic (6 m/pixel, credit: NASA/JPL. The license terms can be found at pds-imaging.jpl.nasa.gov/portal/mro_mission.html). We produced this figure using Esri’s ArcGIS 10.3 (http://www.esri.com/software/arcgis).

Here, we present the first comprehensive map of the NPLD trough cells (Fig. 1A). Our map reveals extensive trough segmentation, which until now had not been integrated into an understanding of the troughs’ geologic history. The cells, which generally have concave elongate shapes, exhibit two types of stratigraphic exposures: (1) those with layers that are traceable around the complete perimeters of the cell walls, as well as the flanks of interior positive relief features such as mounds, thus forming concentrically layered patterns (Figs. 1B, 2A–C, S4–S8) stratigraphically reconstructed in Fig. 3, and (2) those that display layers only on equator-facing slopes (Figs. 1B, 4, S4, S9).

Our map shows that the concentrically layered outcrops within trough cells are common across the entire polar plateau (Fig. 1B, red and blue dots). These stratigraphic exposures, showing traceable layer continuity across cell slopes (Figs. 2B,C, S4–S8), comprise evidence of trough formation and segmentation associated with widespread in-situ NPLD excavation (Fig. 3). Some scarps expose cross-sectional views of this erosional stratigraphy (Figs. S9, S10). Inter-cell ridges of varying lengths and widths, and trough interior mounds, indicate that erosion within troughs occurred heterogeneously (e.g., Figs. 2, S3, S4).

In widespread locations, the equator-facing layers in the concentrically eroded zones extend along the flanks of multiple adjoining cells that lack pole-facing layering (Fig. 4A,B), supporting their shared excavation. We find that the absence of pole-facing strata in most troughs is due to thin mantles covering these surfaces (Figs. 3B, 4C, S6, S8, S9, S10), consistent with previous observations^22^. Hence, the difference between these two trough types (Fig. 1B) reflects a history of recent (possibly ongoing) sedimentary mantling, mostly disconnected from the current troughs’ origin and distribution.

The above observations indicate that in-situ erosion, rather than a reworking of strata due to poleward trough migration, was the primary mechanism for trough development*.* At numerous locations, truncated layers bound the entire relief of trough sections (e.g., Figs. 2B,C, S3, S4). We measured a total volume of ~ 4 × 10^4^ km^3^ for PB troughs (i.e., roughly ten times the volume of the Grand Canyon in Arizona, USA), which we equate to the amount of evacuated NPLD materials (Fig. S11).

Pelletier^21^ suggested that incipient, randomly distributed sublimation troughs migrated poleward until they reached a steady-state, spiral form. This scenario would have led to large-scale reworking of the NPLD due to migration-related mass exchanges. However, our statistical analysis of the outcrops noted in Fig. 1B shows that those with only equator-facing layers mostly fall within ~ 25 km of outcrops with equator- and pole-facing layers (Fig. S12), supporting that trough excavation occurred after NPLD deposition and not within a stratigraphy that was extensively reworked by ice migration.

### Gradual spiral pattern development due to prolonged in-situ erosion

**Figure 5:**
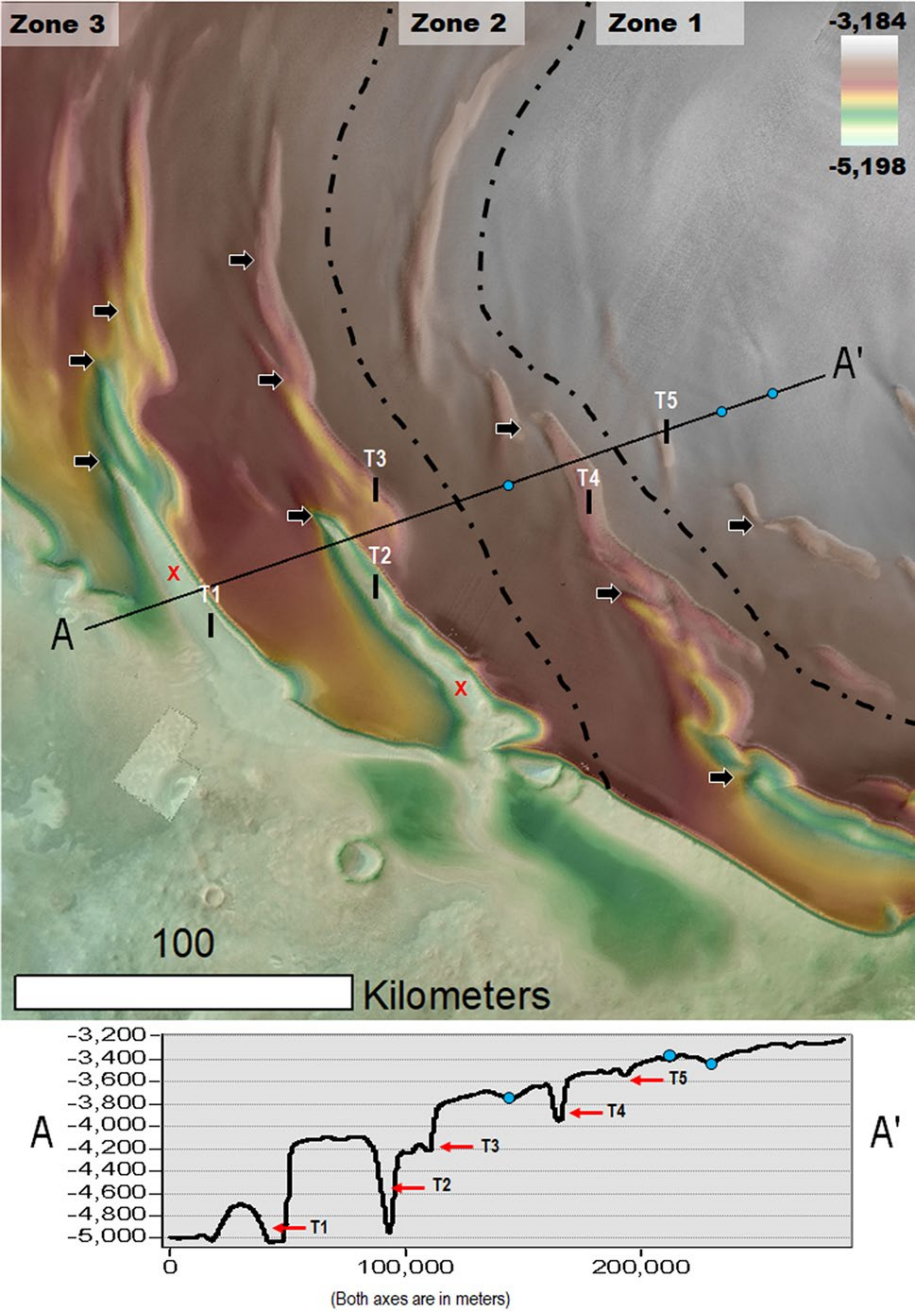
View of PB centered at 81° 38' N, 14° 30' W with a succession of stair-step trough topography (elevation profile), which exhibits increasingly wider, deeper, and more longitudinally extensive spiral arms. Note that we identified the troughs (T1-T5) and undulations (blue dots) situated along the elevation profile’s path. The two red “X” letters identify zones where the trough floors expose the VBF (i.e., the northern plains unit, which in this region underlies the NPLD). Despite the troughs’ topographic and spatial complexity, we find their overall morphologic characteristics reflect varying degrees of development, which broadly fit within our proposed three zonal categories: (1) PB areas where relatively small troughs form sparse clusters (Zone 1). (2) Areas where trough junctures reflect widespread intersection relationships which are spatially manifested as continuous spiral arms. In these areas, the troughs have floors excavated within the NPLD (Zone 2). (3) Areas where these spiral arms are longer and deeper, are closely spaced, and expose geologic surfaces that underlie the NPLD. In the context of this figure, the exposed surfaces are part of the VBF (Zone 3). The dashed lines demark the contacts between Zones 1 & 2 and 2 & 3. All these zones retain a common baseline morphologic attribute – the preservation of inter-cell ridges (black arrows). This observation is consistent with these spatial zones, broadly reflecting the temporal sequence of trough development and spiral pattern generation. In Fig. 6, we graphically present this proposed protracted history of cell enlargement, intersection, and merging leading to the formation of the spiral arms. Our reconstruction in Fig. 6A is based on the spiral arm connecting troughs T2 and T3. We produced this figure using Esri’s ArcGIS 10.3 (http://www.esri.com/software/arcgis).

Within the troughs, the presence of cells, inter-cell ridges, and mounds attest to a history of heterogeneous NPLD excavation. Here, we propose that the connection between this history and the emergence of the spiral pattern is retained in the spatial relationships that can be broadly categorized as (Fig. 5): (1) clusters of small troughs (generally < 100 km in length), in which individual troughs are poorly aligned and relatively distantly spaced. (2) Locally discontinuous spiral arms that are entirely excavated into the NPLD. These arms retain numerous inter-cell ridges and widespread arcuate cell margins. (3) Spiral troughs without significant gaps with floors that exhume surfaces situated beneath the NPLD. These arms retain relatively fewer arcuate cell margins and, in their NPLD excavated segments, exhibit some inter-cell ridges.

**Figure 6:**
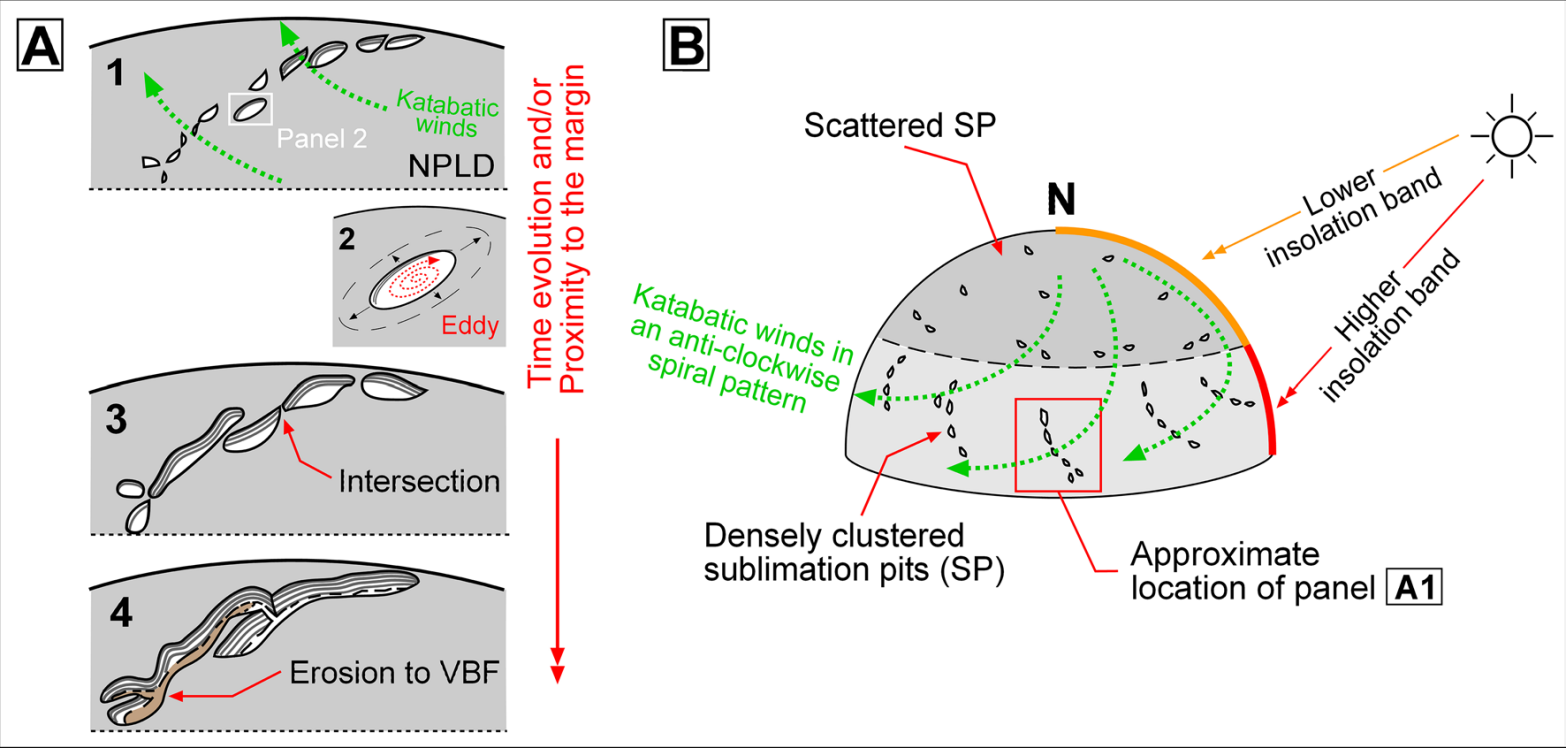
Sketches showing a geologic scenario that explains the formation of the north polar spiral troughs. Our model departs from the assumption that after the NPLD accumulated, PB had a smooth overall domical shape. Solar heating produced widespread sublimation pits (**A1**,**B**), predominantly clustered along the polar plateau’s periphery due to higher insolation (**B**). The sublimation pits gradually grew obliquely to the direction of katabatic winds, which have flow directions  patterned in an anticlockwise spiral. The apparently enhanced erosion linked to lateral wind dispersion might have been related to the development of powerful wind vortices (**A2**,**A3**). As the troughs grew and deepened, their intersections increased, leading to the emergence of spiral arms, which, while mostly dissected within the NPLD, locally expose underlying geologic units (**A4**). The spiral arm shown in panel A3 is a reconstruction of that which includes troughs labeled T2 and T3 in Fig. 5.

**Figure 7:**
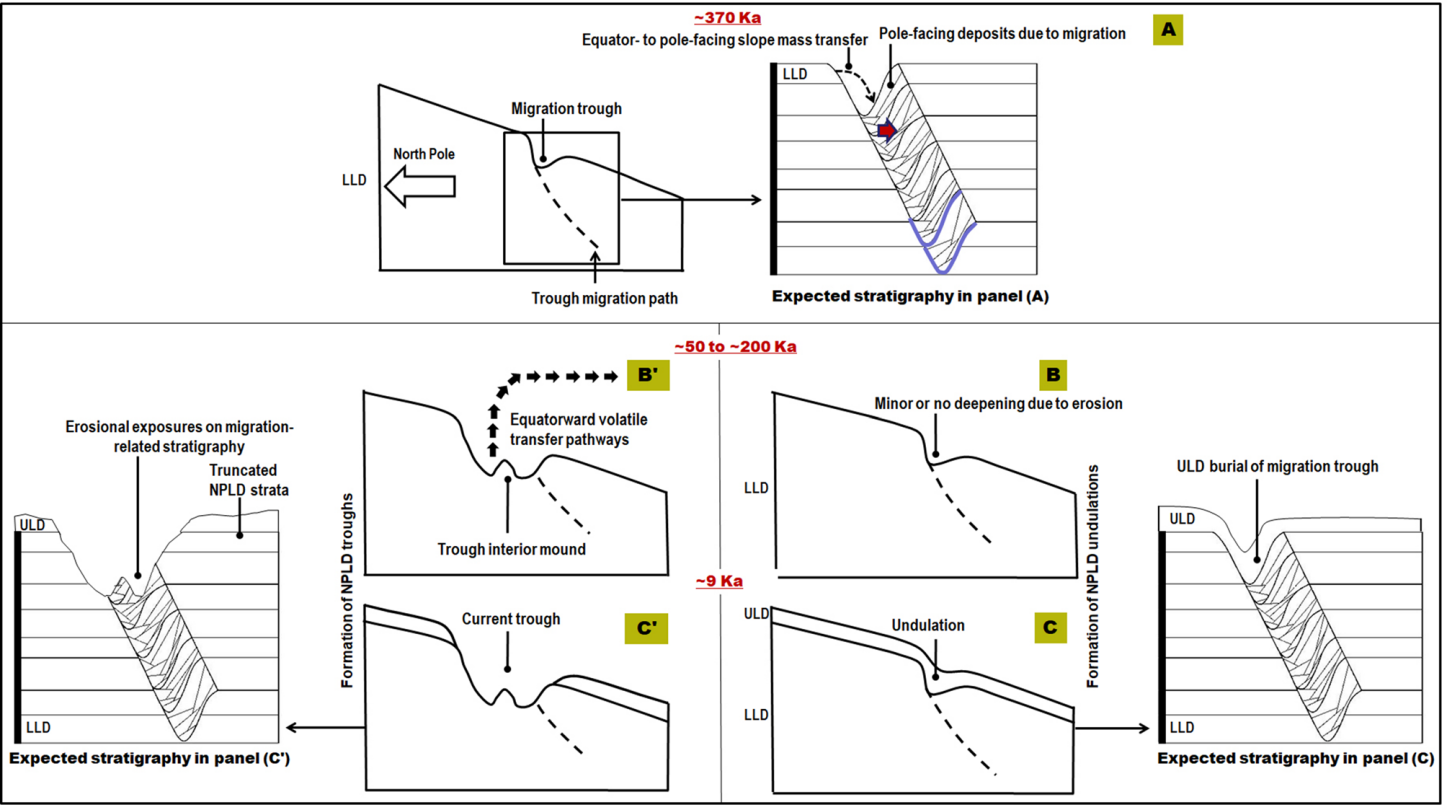
Sketches depicting how poleward trough migration could have shaped the NPLD stratigraphy associated with undulations (**A**–**C**), and trough interior stratal truncation morphologies (**A**–**C'**). (**A**) Following Mars’ last ice age ~ 370 Ka as proposed by Smith et al.^13^, polar ice accumulation during low mean obliquity led to the emplacement of the top few hundred meters of the LLD, concurrently with the poleward migration of troughs, which resulted in the formation of radar detectable migration pathways. The indigo lines trace buried, paleotrough surfaces, expected to have remained preserved within the NPLD as the trough migrated. Above these paleosurfaces, we expect to find cross-bedding (red arrow) forming cross-sectional stratal geometries resulting from sustained deposition on the inclined, migrating, pole-facing slopes. (**B**,**B'**) A geologically recent phase of surface erosion, probably ~ 50–200 Ka^28^, resulted in significant, heterogeneously distributed, polar ice removal. Panel (**B**) shows a migrating trough that experienced no or little erosional deepening during this stage. Panel (**B'**) shows a migrating trough that experienced significant erosional deepening during this period. (**C**) The ULD were emplaced ~ 9 Ka^1,7^ and buried some troughs, topographically muting them and resulting in the formation of the undulations. The proposed migration ceases at this point. (**C'**) The ULD are emplaced ~ 9 Ka^1,7^ and retained on the trough surroundings but appear to have heterogeneously retreated from inside the eroded troughs, or alternatively, the trough interiors lacked a favorable depositional environment for the ULD. The expected stratigraphy sketches shown in this figure comprise predictions that need to be validated through comparison of our observations to those on which the proposed cross-sections for scenarios of poleward migration were based on (-also see Fig. 3c in Smith and Holt^16^ and Fig. 5 in Smith and Holt^19^-).

We hypothesize that the following sequence of events links the troughs’ formational history to the spiral pattern’s emergence (Fig. 6). Following the LLD emplacement and the establishment of the current overall PB topography, sublimation pits developed over a generally smooth, undissected surface. There could also have been an older pre-existing population of topographically subtler migrating troughs (Fig. 7).

Spiga and Smith^23^ performed a wind field simulation over a significant portion of PB (see their Fig. 5) using the “Laboratoire de Météorologie Dynamique” (LMD) Martian Mesoscale Model (MMM)^24,25^. Their grid spacing and horizontal wind vectors were 6.7 km and 50 m, respectively. The simulated wind patterns reveal flow trends that typically exist orthogonally or obliquely to the troughs. Hence, our conceptual model  assumes that winds did not generally flow along the troughs, but across them.

Troughs generally integrate multiple cells that are typically elliptical or elongate, which include widespread mounds and exhibit floors without evidence of unidirectional wind erosion (e.g., ridges and grooves transverse to the troughs). We attribute the elongate cell shapes to erosion by wind vortices (panel A2 in Fig. 6) which generally developed laterally to katabatic wind flow directions over the troughs. The growth and merging of widespread cells generated the segmented troughs, which progressively coalesced to form the spiral pattern. While further computer simulation of wind patterns tailored to high-resolution PB topography is required to validate this concept, this hypothetical scenario has support in large-eddy simulations that find that counter-rotating helical vortices played a crucial role in excavating moats, which at widespread locations on Mars, surround crater interior sedimentary mounds (e.g., Gale crater)^26,27^.

Our proposed geologic scenario implies a landscape evolution in which pits developed into cells, and cells progressively merged into troughs. While most of the troughs would have interlaced through vertex-to-vertex interconnections (explaining their typical longitudinal continuity), vertex-to-midsection intersections produced widespread forking patterns (e.g., Fig. S7). As long chains of troughs became joined, the regional spiral pattern developed. Our model resolves that the trough spiral pattern is an in-situ erosional spatial byproduct, and therefore did not result from the poleward migration of initially randomly distributed troughs^21^.

The shallowly eroded concentric layers exposed along many cell vertices (e.g., Figs. 2, S6, S8) indicate cell growth occurred within the uppermost surface and near-surface NPLD stratigraphy, and not into a pre-existing troughed and reworked topography. Hence, the hypothesized pits/migrating troughs from which the cells could have initially nucleated probably were widely spaced apart, consistent with a progressive history of trough growth into the spiral arms.

Due to the polar plateau’s domical shape, the extent of equator-facing surfaces (and hence the expected magnitude of insolation-driven sublimation) increases with distance from the pole. Possibly, the erosive power of wind vortices that developed within the pits and growing cells also increased near the plateau’s margins due to increases in slope and entrainment of fines. Consequently, while the sublimation pits’ initial distribution was probably random, their subsequent growth would have been greater closer to PB’s periphery, where the troughs are most developed and best integrated.

### Evidence of widespread buried troughs

**Figure 8:**
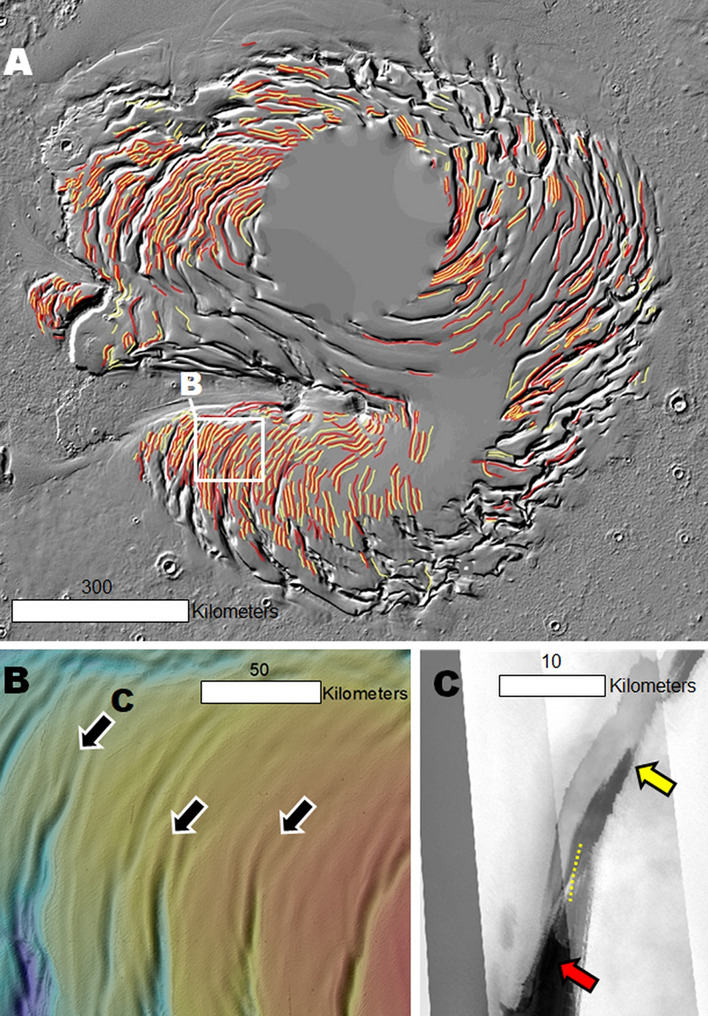
(**A**) Map showing the undulations of PB. The yellow lines trace the floors and the red lines trace the adjoining ridges. The base map is a MOLA shaded relief, illumination from the upper left (256 pixels/degree, credit: MOLA Science Team, MSS, JPL, NASA). (**B**) A close-up view shows troughs connected to undulations (black arrows show nexus points). The image is a color MOLA digital elevation model draped over a MOLA shaded relief; illumination from the upper left (256 pixels/degree, credit: MOLA Science Team, MSS, JPL, NASA). (**C**) View of dark lithic materials at the nexus between a trough (red arrow) and an undulation (yellow arrow). The dashed line identifies the contact between the undulation and the trough. The panel is part of a CTX mosaic (6 m/pixel, credit: NASA/JPL. The license terms can be found at pds-imaging.jpl.nasa.gov/portal/mro_mission.html). We produced this figure using Esri’s ArcGIS 10.3 (http://www.esri.com/software/arcgis).

We produced the first comprehensive map of the PB undulations. The map reveals that the undulations follow and extend the troughs’ clockwise spiraling pattern, particularly on upper plateau surfaces (Fig. 8A). Locally, they transition into troughs (Fig. 8B,C). Undulations thus may be the surface expressions of relatively narrow and shallow trough sections that are now entirely buried by the ULD. In this scenario, before the ULD emplacement, a large subset of these older troughs experienced an episode of erosion, coalescence, and enlargement along their original pattern. This phase of erosion could have led to the development of the stratal truncation morphologies that we observe within the troughs (Figs. 1B, 2A–C, S4–S8). Hence, the total volume of excavated NPLD ice during trough development could exceed our ~ 4 × 10^4^ km^3^ estimate. A consequence of this geologic scenario is that the spiral pattern must have also developed before ULD emplacement.

## Discussion

### Constraints on the timing of NPLD trough formation

Our interpretation of the undulations as ULD-buried trough sections implies that the troughs developed largely after the accumulation of the LLD^1,7^, and before ULD deposition ~ 10 Ka^7^. The age of the LLD trough strata remains uncertain, with estimates ranging from ~ 3.6 ± 2.5 Ma^7^ to ~ 370 Ka^13^. The younger age bracket for trough development (between ~ 370 Ka and ~ 10 Ka) suggests that trough development could represent evidence for one of the most recent episodes of climate change on Mars. There is additional evidence of potential warmer paleoclimates slightly more recent than ~ 370 Ka. For example, the migration of aeolian bedforms in the Meridiani region ~ 50–200 Ka^28^ is interpreted as an indicator of a significant increase in Mars’ atmospheric density and pressure. There are also possible aqueous debris flows from ∼ 190 Ka^29^ in Istok crater (45.11° S, 274.2° E), a very young crater in Aonia Terra. Perhaps the water ice removal recorded in the uppermost sequences of the NPLD exposures along troughs’ walls and floors was coeval with these recent paleoclimate events.

Conversely, if the LLD surfaces exposed within the troughs are ~ 3.6 ± 2.5 Ma^7^, the geologic scenario arises that trough excavation might have been a cumulative process spanning numerous high-obliquity cycles. In this case, the larger troughs, mostly along the PB periphery, would have likely been exposed to more intense and reoccurring resurfacing during these cycles. Hence, they might be sites where alternating erosion and deposition more frequently took place during trough excavation, particularly in areas where NPLD sediments were remobilized and not completely removed from these larger troughs. Evidence of these intercalated inner-trough erosional depositional cycles is preserved as widespread unconformities mapped in some of these larger trough sets^7^.

### Trough excavated ice as a source of the mid-latitude mantles

Three-dimensional Global Climate Models [GCMs] indicate that PB water ice sublimation^3^ during periods of high mean obliquity (> 35°) transported enormous water vapor volumes to lower latitudes^3,5,8^. Recondensing water vapor likely formed widespread lower latitude icy deposits, including the extensive Latitude Dependent Mantle (LDM)^30^, plains regions with evidence of excess ice^31–34^ and glaciers at mid-latitudes^35,36^. Moreover, this atmospheric and paleoclimatic cyclicity is thought to have also generated tropical mountain glaciers^8,37^. However, thus far, these atmospheric models face a significant handicap —  their implied large-scale NPLD erosional events, which led to the equatorward water vapor transport, have not been successfully reconciled with direct observational evidence in the exposed stratigraphy. The evidence to support these events was limited to the documentation of unconformities inferred from theoretical stratigraphic reconstructions^3^ and radar subsurface detections^13^.

Our proposed geologic scenario fills this gap by interpreting the entire spiral PB troughs as erosional landforms. We estimated that trough excavation could have removed ~ 4 × 10^4^ km^3^ of water ice from PB (Fig. S11). Comparing this result to the estimate of the LDM volume (~ 1 m Global Equivalent Layer (GEL)^38^, corresponding to ~ 1.4 × 10^5^ km^3^), leads us to conclude that upwards of ~ 25% of the ice deposited at non-polar latitudes to form the LDM could have originated from the ice lost in the polar erosional event we propose here. This approximation does not include the potentially coeval retreat of a circum-polar ice sheet, which Tanaka^7^ reconstructed based on the mapping and characterization of numerous NPLD outliers, nor from a potential mass of eroded volatiles from the ULD-buried troughs that now form the undulations, as hypothesized above.

### Significance for future human exploration

The assumption of the recent timeline (e.g., ~200–50 Ka) for the polar erosional event carries potentially significant implications for future human exploration. Following the proposed water vapor deposition at lower, non-polar latitudes, these young deposits would not have existed sufficiently long to experience large obliquity excursions. Hence, they would have remained wholly frozen during their entire post-depositional history, avoiding possible episodic interstitial thawing and refreezing such as that associated with wet-based glaciation^31,36^. Transient melting would have dissolved and increased within these materials the concentration of perchlorates, a widespread Martian surface compound and toxic to humans^39^.

Hence, these young “clean” ice deposits could be an essential resource for future human explorers. We estimated that ~ 40,000 km^3^ of water ice were removed during trough growth, which is volumetrically equivalent to 16 billion Olympic pools. Drinking three liters a day would take 2283 years for a single person to drink an Olympic-sized pool.

 Additionally, the deposits’ youthfulness implies that they likely formed widespread near-surface stratigraphy, enabling easy access. Such deposition may account for the relatively pure water ice beneath a centimeters-thick soil cover discovered by the Phoenix lander in the high latitudes of the northern plains^40^ or shallowly buried water ice within the upper meter of the Martian surface at the mid-latitudes^41,42^.

## Synthesis of trough observations: general constraints on their mode of formation

We propose that future investigations regarding the origin and evolution of the NPLD troughs need to account for the following observations and related interpretations:

1. Stratigraphy in trough walls reveals that layering is horizontal and occurs evenly, with uniform thicknesses, in the plateau wall all around the troughs. Hence, the bulk of the exposed stratigraphy consists of layers that were emplaced during the construction of PB.
2. The mantles on pole-facing slopes (Figs. 4, S6, S8, S9) are mostly disconnected from the current troughs’ origin and distribution.
3. Trough, undulation, and layer geometries indicate in-situ erosion on the LLD surface, below the ULD. Troughs tend to be deeper, wider, and more connected toward the margins of PB, where some trough floors expose basal unit and Vastitas plains materials. Hence, their in-situ excavation might have been enhanced throughout the more steeply sloping areas of PB, probably due to increased insolation on these areas when compared to the flatter, and more central (hence higher latitude), parts of the polar plateau (Fig. 6).
4. The cells and troughs exhibit complex intersections from which their spiral distribution emerges, suggesting that the pattern is a late-stage consequence of the troughs’ erosional history. The troughs’ growth patterns reflect an overall growth trend in directions orthogonal or oblique to regional katabatic winds, suggesting a possible morphogenetic connection.
5. The troughs largely ceased developing before ULD accumulation, given that apparently these materials completely filled some of their sections (i.e., undulations). Hence, Mars’ current and recent climate does not include the atmospheric conditions that led to trough formation and the development of the spiral pattern.

## Conclusions and major remaining issues

Our geologic investigation reveals widespread segmentation of the north polar troughs, which most likely resulted during geologically recent in-situ erosion. The inferred growth trends are consistent with the erosion occurring preferentially orthogonally and obliquely to katabatic wind directions and suggest that the spiral pattern is a consequence of the troughs’ progressive spatial integration. Furthermore, the spiral pattern’s connection to landforms indicative of cell and trough intersections implies that there has been no appreciable repositioning of the trough axes subsequent to (and during) the emergence of the spiral arrangement. Our hypothesis explains that the troughs’ spiral trends developed over the plateau’s current topography. A similar in-situ erosional history was also likely responsible for the origin of the south polar spiral troughs^43,44^.

Major uncertainties and controversies that remain unresolved include:

1. A non-migratory nature of the polar troughs implies that the strata that they expose on both their equator and pole-facing slopes can be linked to paleoclimatic conditions accompanying the construction of the Martian polar plateaus. If, as previously proposed^1^, the NPLD contain layers deposited during the last billion years, then formation of the troughs could have exposed a paleoclimatic record spanning about a fourth of the planet’s geologic history. If on the other hand, the NPLD formed during the last 4 Myrs^3^, the exposed layers likely comprise an extensive paleoclimatic record of the geologically recent evolution of the Martian paleoclimate.
2. SHARAD subsurface radar data characterizations in Smith and Holt^16,19^ show possible trough migration paths beneath some of the undulations. If the undulations present within these areas are indeed buried migratory troughs, a reasonable  prediction is that  their migration paths would (1) trace a shrinking spiral (if the migrating troughs’ initial distribution was more peripheral and spiral), or (2) the pathways that led to the emergence of the spiral pattern (if their original distribution was randomized^21^). However, due to the orbital inclination of the Mars Reconnaissance Orbiter, the innermost areas of PB lack observations, which makes these determinations challenging.
3. The interpretation that inclined radar discontinuities are trough migration paths is partly based on the observation that their uppermost zones terminate right beneath trough floors. However, it remains unclear whether other geologic scenarios could explain the radar-reconstructed stratigraphy. For example, some of these could be buried unconformities, which in the past surfaced at breaks-in-slope (Fig. S13). Troughs could have preferentially developed on the slope breaks due to enhanced erosion (perhaps due to preferential dark lithic mantling, probably dust). The formation of these troughs would have resulted in virtually the same NPLD stratigraphy as that documented to support the migration scenario. Further testing of this hypothesis includes (1) determining whether these other types of unconformities could be present in at least a subset of the troughs, (2) identifying if there are any radar signatures similar to the proposed migration paths that extend to the surface with no associated trough.

## Supplementary Information

Supplementary Information
