# Supplementary material for: North polar trough formation due to in-situ erosion as a source of young ice in mid-latitudinal mantles on Mars
Source: Sci Rep. 2021 Mar 25;11:6750. doi: 10.1038/s41598-021-83329-3 (PMC7994824; doi:10.1038/s41598-021-83329-3)
Supplement: Supplementary file 1 — Supplementary Information [file 41598_2021_83329_MOESM1_ESM.docx]

**North Polar Trough Formation Due to In-Situ Erosion as a Source of Young Ice in Mid-Latitudinal Mantles on Mars**

J. Alexis P. Rodriguez^1*^, Kenneth L. Tanaka^1^, Ali M. Bramson^2^, Gregory J. Leonard^3^, Victor R. Baker^3,4^, and Mario Zarroca^5^

*^1^Planetary Science Institute, 1700 East Fort Lowell Road, Suite 106, Tucson, AZ 85719-2395, USA.*

*^2^Department of Earth, Atmospheric, and Planetary Sciences, Purdue University, 550 Stadium Mall Dr., West Lafayette, IN 47907*

*^3^Department of Planetary Sciences, Lunar and Planetary Laboratory, University of Arizona, Tucson, AZ 85721, USA.*

*^4^Department of Hydrology & Atmospheric Sciences, University of Arizona, Tucson, AZ 85721, USA.*

*^5^External Geodynamics and Hydrogeology Group, Department of Geology, Autonomous University of Barcelona, 08193 Bellaterra, Barcelona, Spain.*

*Correspondence to alexis@psi.edu

**Supplementary Figures**


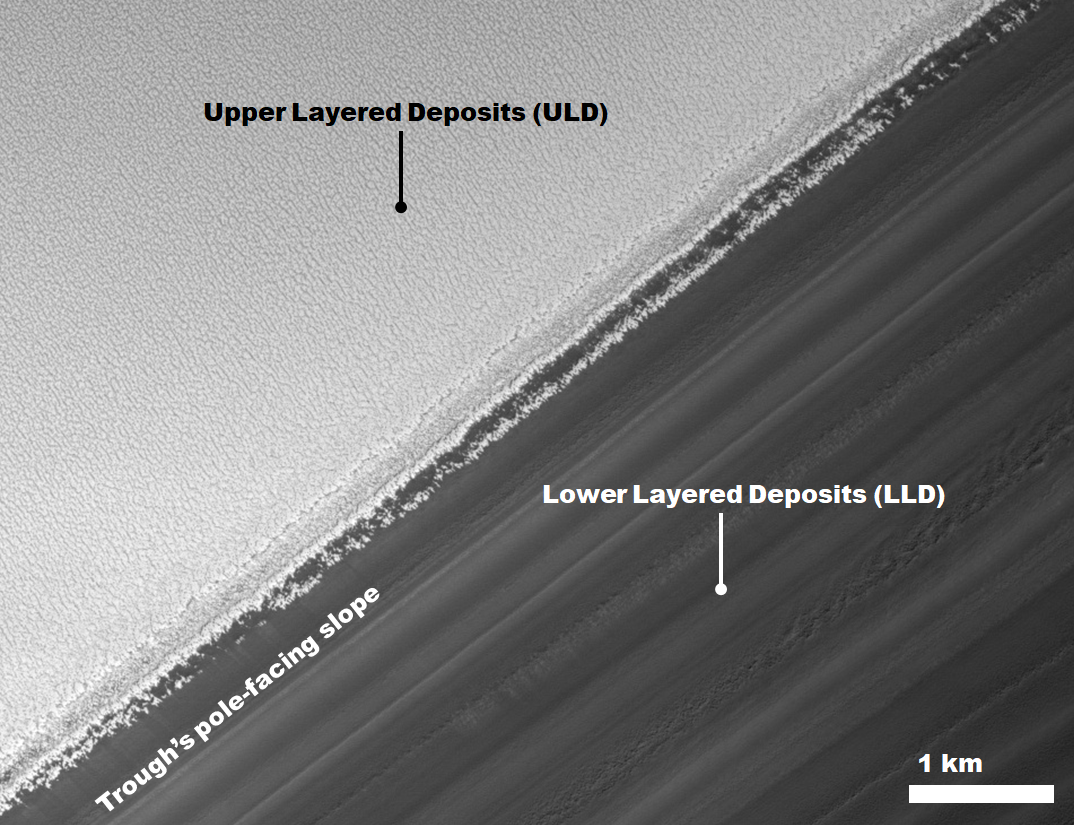
**Figure S1** Part of HiRISE ESP_019317_2640 image centered at 83°49' N, 64°13' E. The view shows the contact between the ULD and LLD deposits at and near the margin of a trough pole-facing slope. The ULD are also mostly covered by a bright, coarsely textured, decimeters to meter-thick residual ice cap. We produced this figure using Esri’s ArcGIS 10.3 (<http://www.esri.com/software/arcgis>).


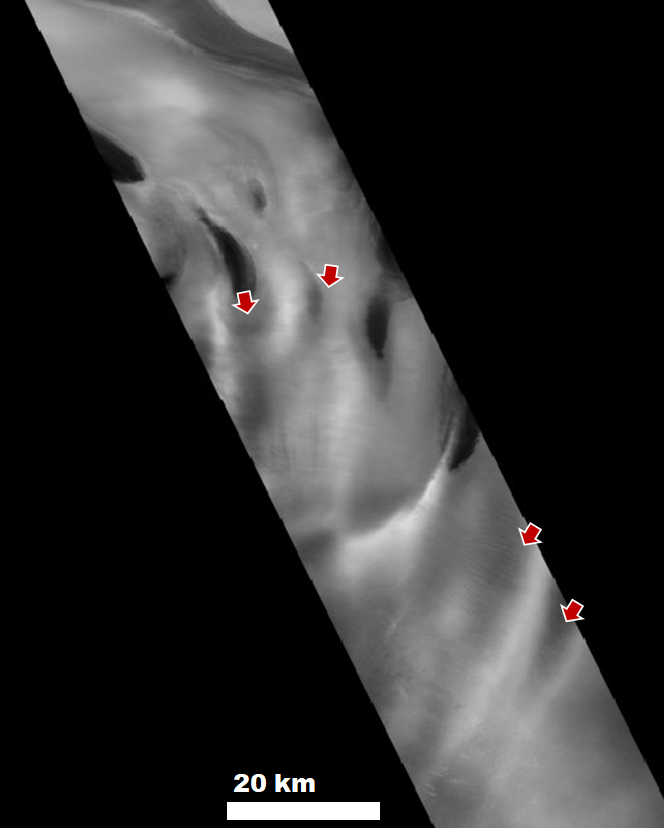


**Figure S2** CTX view centered at 84°00' N, 12°90' E showing surface undulations (directions of red arrows). We produced this figure using Esri’s ArcGIS 10.3 (<http://www.esri.com/software/arcgis>).

**Figure S3** High-resolution MOLA topography (512 pixels per degree) draped over a CTX mosaic (~7 m per pixel) centered at 85°10' N, 110°59' W. The dark lines are surface contours that trace constant elevations and which illustrate the locations of enclosed depressions (cells). Cells labeled A-E are at least partly bounded by trough inner ridges within the troughs (e.g., red arrows). Those labeled 1-3 are mostly nested between the trough’s pole-facing and equator-facing slopes. We produced this figure using Esri’s ArcGIS 10.3 (<http://www.esri.com/software/arcgis>).


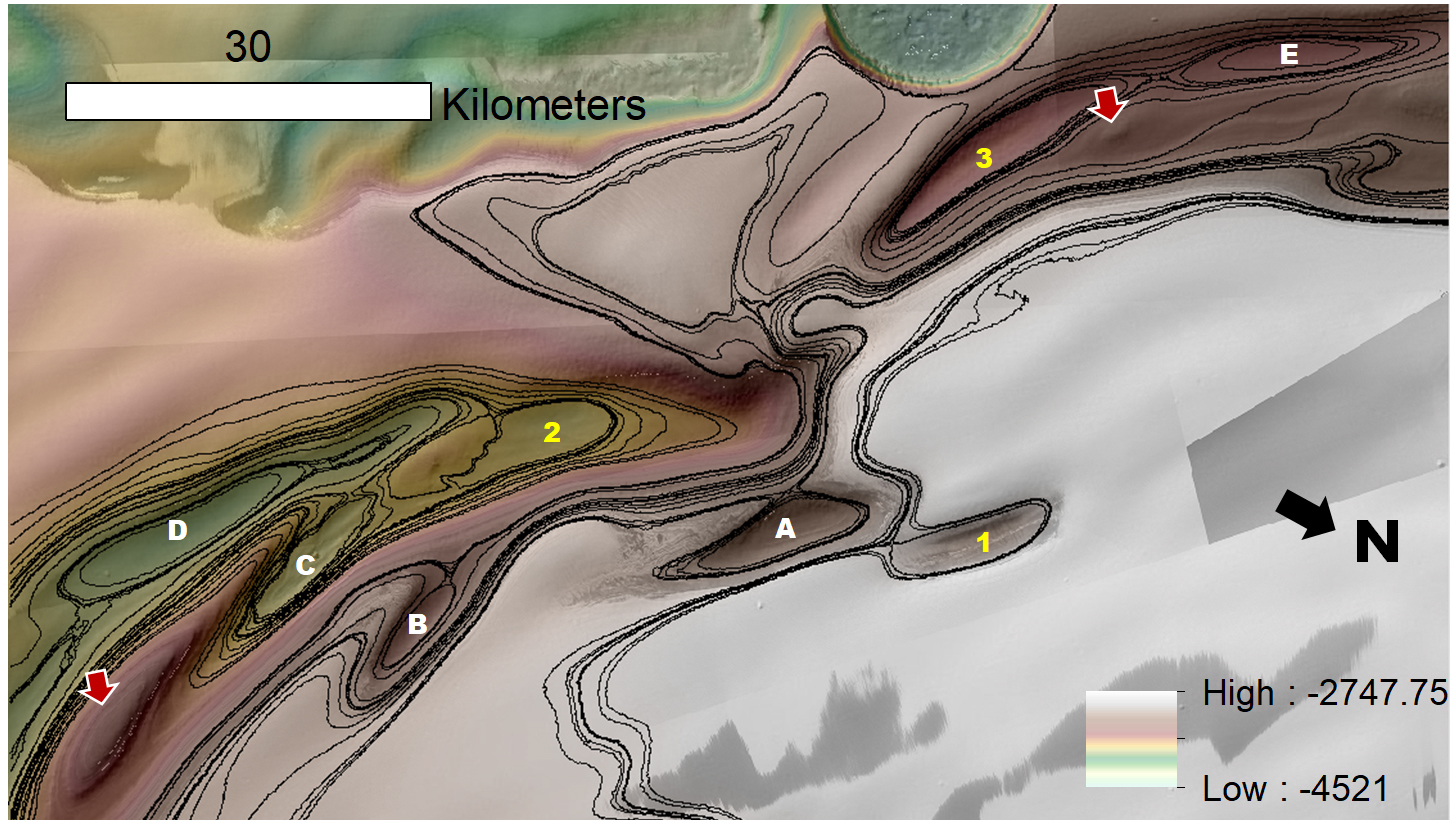


**Figure S4** CTX view underlying MOLA topography of an NPLD trough centered at 86° 52' N, 24°80' W. The image includes five distinct cells and a large mound (all labeled). The white arrows identify pole-facing layers that transition along the trough’s axis into pole-facing sections that lack the layers (red arrows), probably because mantles obscure their exposures. The evidence of truncation permits us to reconstruct an approximate pre-erosion paleo-surface above the elevation profile section that crosses the trough (red line in lower profile). We produced this figure using Esri’s ArcGIS 10.3 (<http://www.esri.com/software/arcgis>).


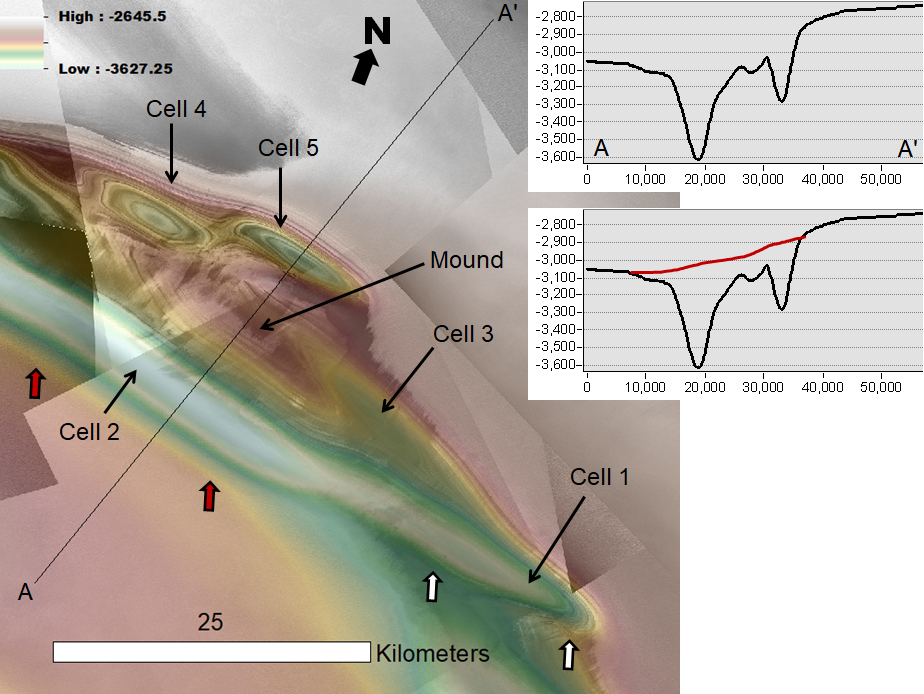


**Figure S5** **(A)** Context and locations for panels (B-D). The base map is a MOLA shaded relief, artificially illuminated from the upper left (256 pixels/degree, credit: MOLA Science Team, MSS, JPL, NASA). **(B-D)** Views of trough sections that exhibit morphologic and morphometric characteristics similar to those in Fig. 2A. For example, The red arrows identify equator and pole-facing strata that bound extensive sections of the troughs’ interiors. The outcrops are far apart from each other, and hence complement Fig. 1B by showing that these types of trough erosional morphologies are typical. The panel centers are (B, 84°36' N, 136°47' E; C, 82°54' N, 53° 40' E; D, 80°10' N, 5°54' W). Panels **(B-D)** are parts of a CTX mosaic (6 m/pixel, credit: NASA/JPL. The license terms can be found at pds-imaging.jpl.nasa.gov/portal/mro_mission.html). We produced this figure using Esri’s ArcGIS 10.3 (<http://www.esri.com/software/arcgis>).


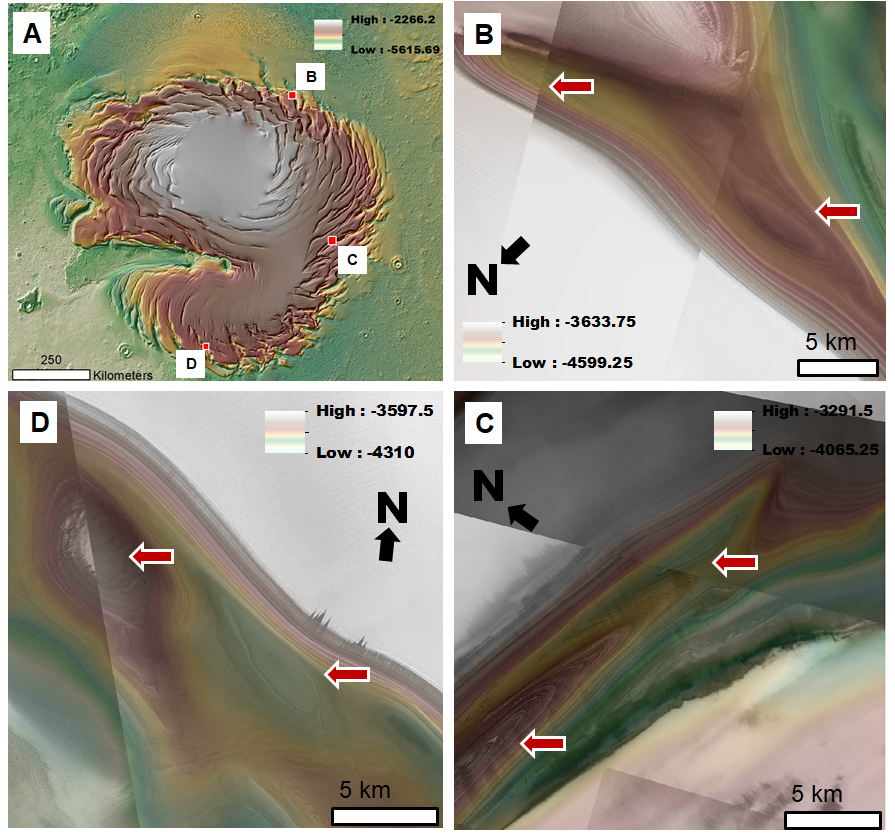

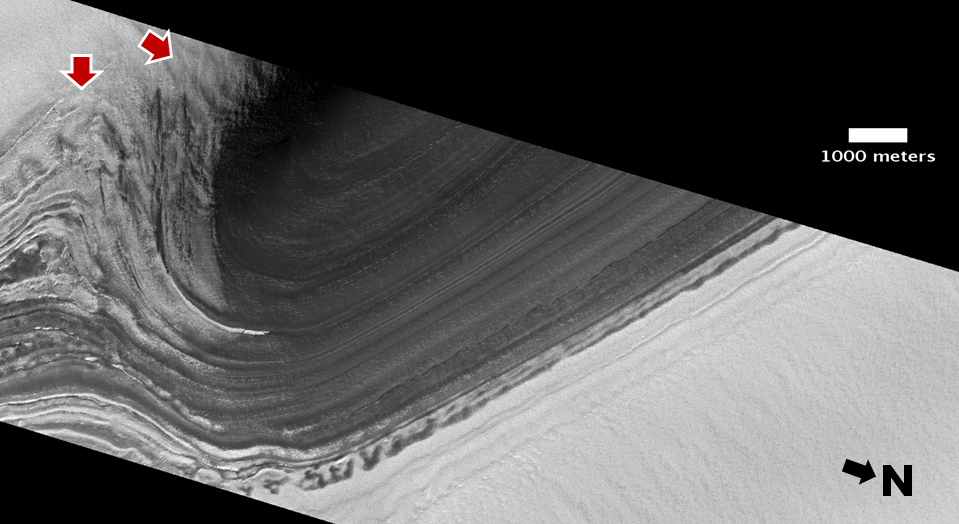


**Figure S6** Part of HiRISE image ESP_018810_2655 centered at 85°50' N, 117°15' W. The red arrow identifies pole-facing flanks where concentric layers that are covered but not completely obscured by mantling. These observations suggest that pole-facing slopes preserve evidence of truncation, which has been extensively obscured by later deposition. This image was obtained during the northern summer (https://hirise.lpl.arizona.edu/ESP_018361_2655). We produced this figure using Esri’s ArcGIS 10.3 (<http://www.esri.com/software/arcgis>).


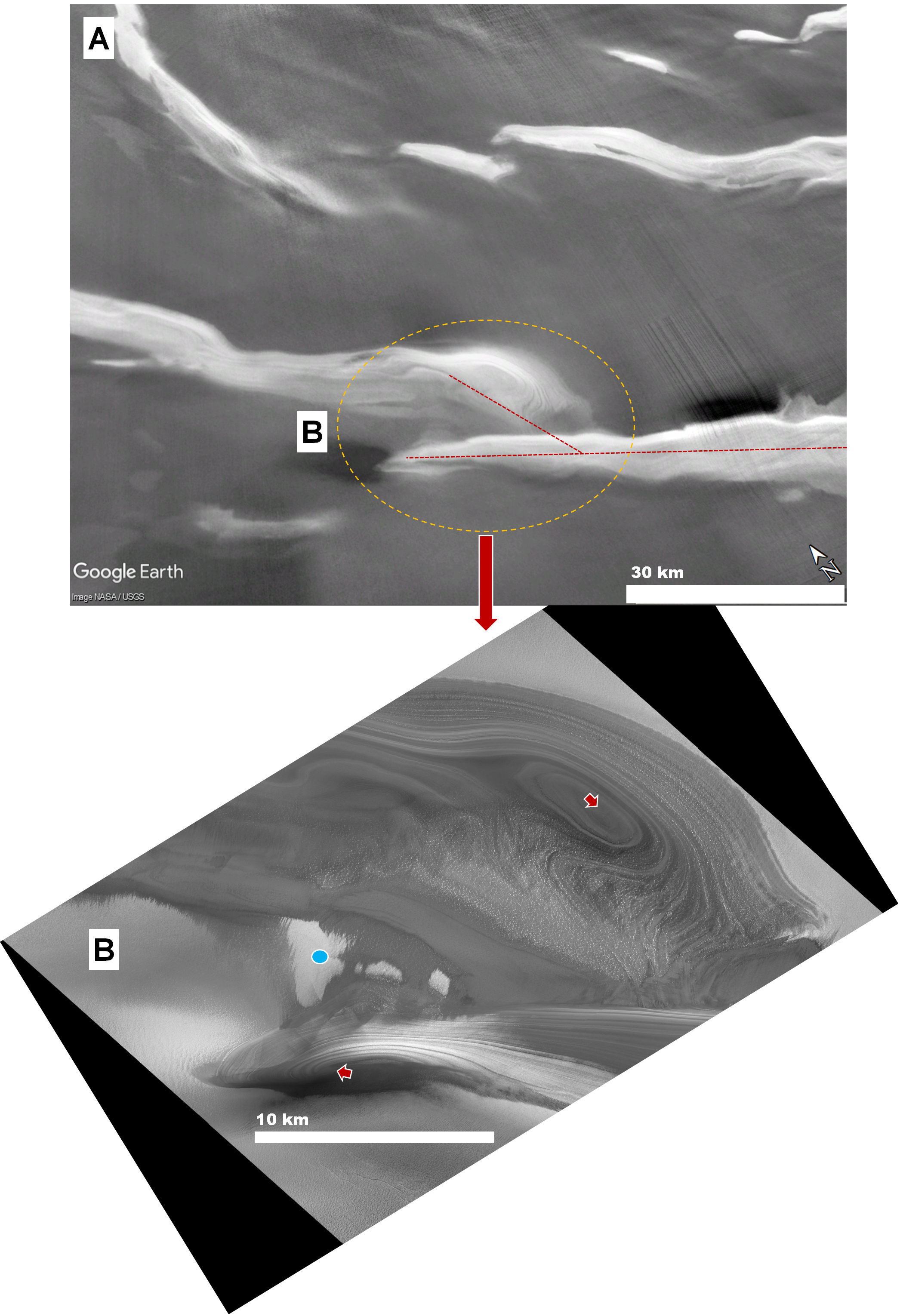


**Figure S7 (A)** View of THEMIS daytime IR mosaic centered at 80°14' N, 15°30' W, in which a forking pattern between two troughs is indicated by the dashed red lines. **(B)** CTX close-up on the trough juncture at the forking zone. The red arrows show how each trough terminates in a cell and the blue dot shows the intertrough NLPD surface. The intersection between these troughs contributed to the growth and integration of their broader spiral pattern. We produced this figure using Esri’s ArcGIS 10.3 (<http://www.esri.com/software/arcgis>).

**Figure S8** CTX view centered at 81°37' N, 68°47' E showing concentric layers exposed by in-situ NPLD truncation. The dashed red arrows trace these layer sequences up to the base of the ULD deposits. Localized unconformities at the cell’s end (red arrow) show that the erosion alternated with some degree of deposition, perhaps because the in-situ erosional debris remained within the troughs. This image was obtained during the northern summer (http://viewer.mars.asu.edu/planetview/inst/ctx/G02_019027_2619_XN_81N291W#P=G02_019027_2619_XN_81N291W&T=2). We produced this figure using Esri’s ArcGIS 10.3 (<http://www.esri.com/software/arcgis>).


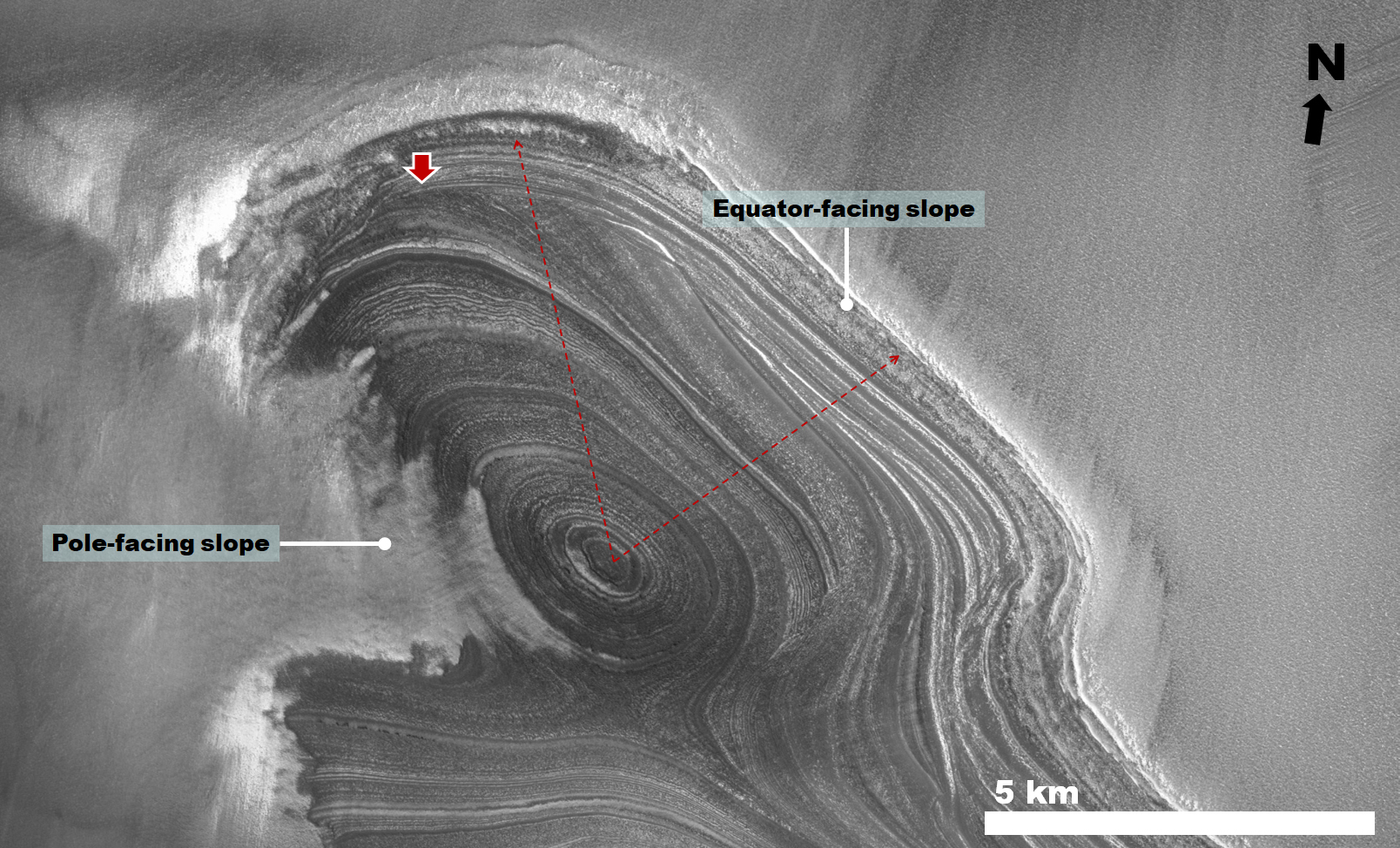

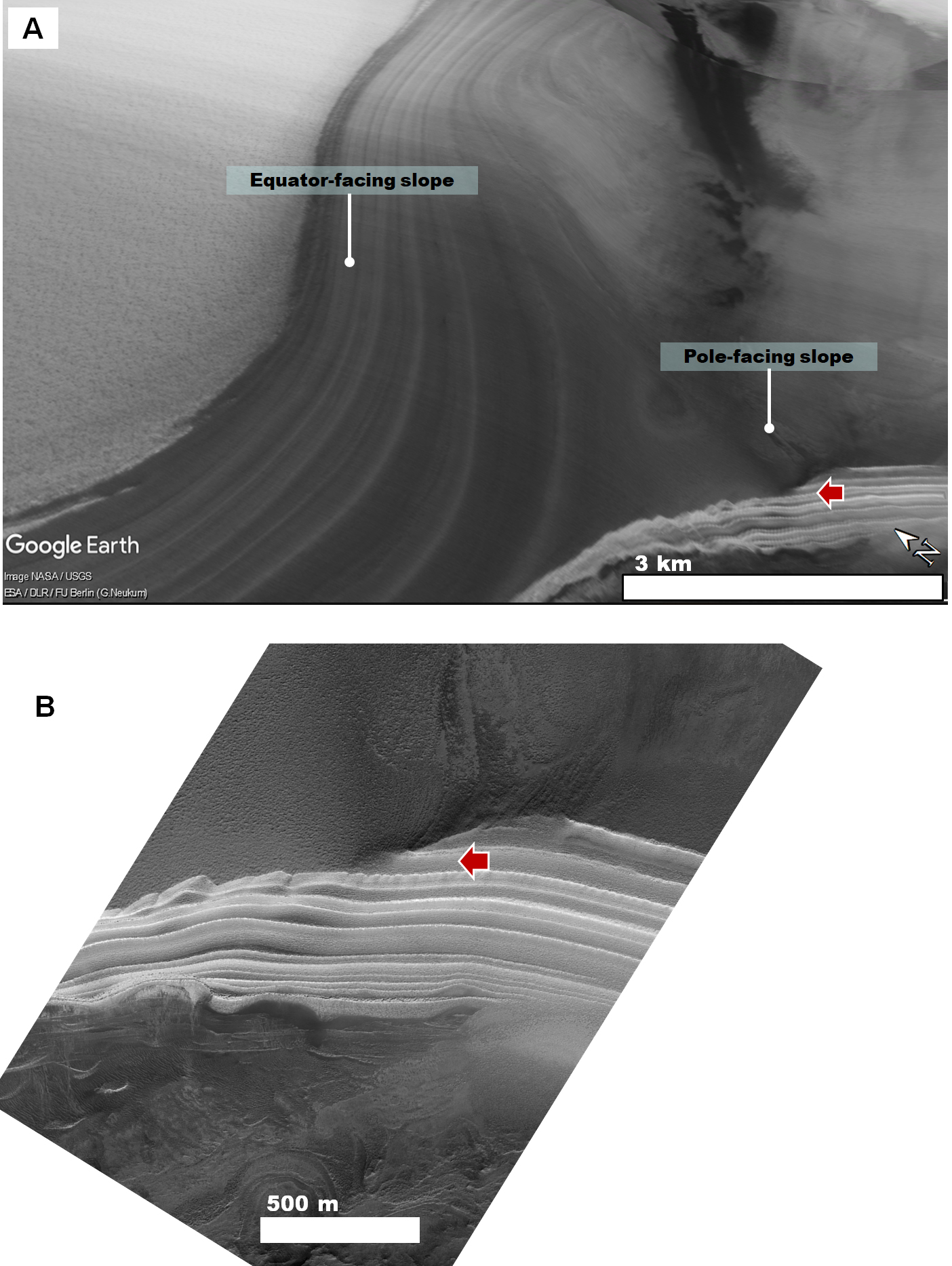


**Figure S9 (A)** CTX perspective view of a trough centered at 85°20' N,153°20' E. Notice that the trough’s pole-facing slopes lack widespread layered exposures. A scarp reveals, however, evidence of truncated layers covered by thin mantles (red arrows). **(B)** Close-up ESP_019274_2650 view on panel (A, red arrows identify the same locations). We produced this figure using Esri’s ArcGIS 10.3 (<http://www.esri.com/software/arcgis>).

**
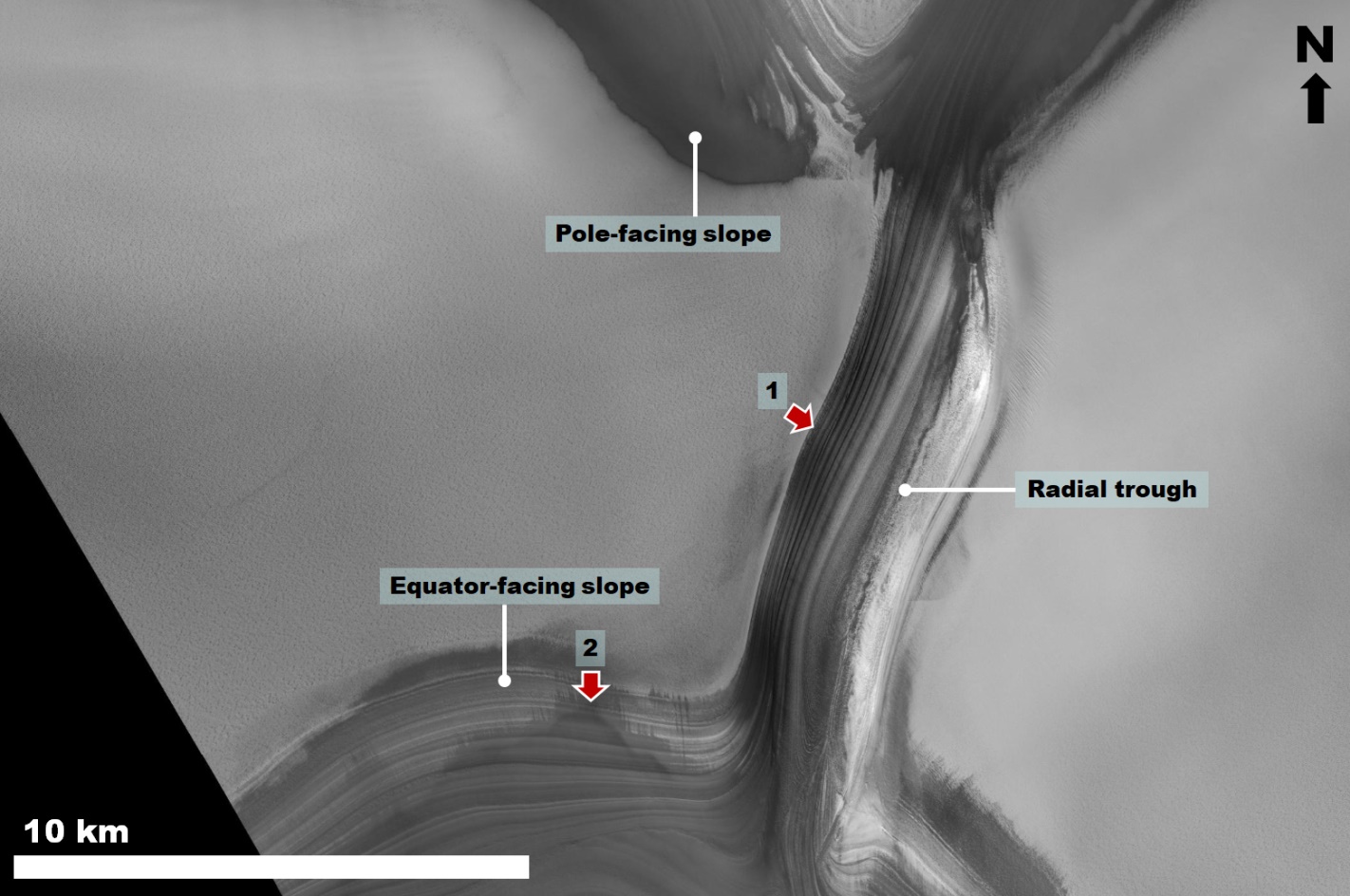
Figure S10** CTX view centered at 84°31' N,104°54' E showing a rare occurrence of a radial trough, which exposes the NPLD stratigraphy situated between the equator-facing and pole-facing slopes of two troughs (red arrow 1). This layer pack is a continuation of those exposed on the equator-facing slopes (red arrow 2). These observations indicate that, at least in this region and to the depths of dissection, the NPLD stratigraphy between the two troughs has not been reworked due to migration. This image was obtained during the northern summer (http://viewer.mars.asu.edu/planetview/inst/ctx/G03_019302_2639_XN_83N250W#P=G03_019302_2639_XN_83N250W&T=2). We produced this figure using Esri’s ArcGIS 10.3 (<http://www.esri.com/software/arcgis>).


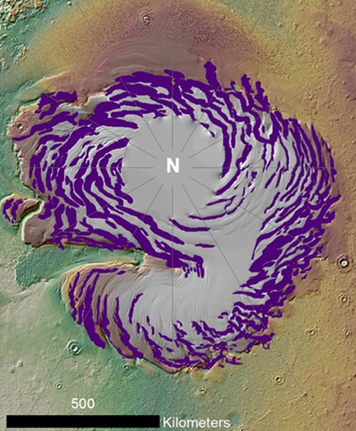


**Figure S11** Purple colored areas show the locations of north polar troughs. We defined buffer polygons around each mapped trough and used elevation points along their rims to produce a triangulated irregular network (TIN). We produced a gridded reconstructed ‘capping’ surface across PB. We subtracted the troughs’ current topography from the modeled capping surface to estimate the approximate erosional volumes consequent of their excavation. The volumes were estimated from MOLA-based digital elevation models (~115 m/pixel with a one-meter vertical accuracy). The base image is a color MOLA digital elevation model draped over a MOLA shaded relief; illumination from the upper left (256 pixels/degree, credit: MOLA Science Team, MSS, JPL, NASA). We produced this figure using Esri’s ArcGIS 10.3 (<http://www.esri.com/software/arcgis>).

**
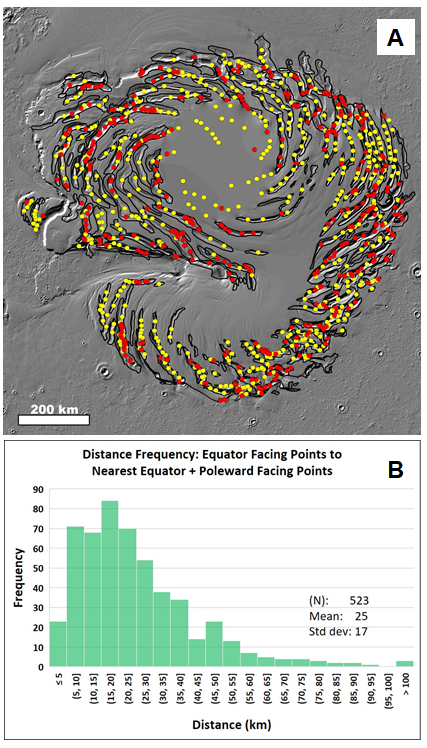
**

**Figure S12** **(A)** Map showing the distribution of topographic cells within the north polar troughs (N = 950). The red and yellow dots mark exposures of interior stratigraphy within the Lower Layered Deposits (LLD) at locations that exhibit both equator and pole-facing layers (red; N=427) and locations where there are only equator-facing layers exposed at the surface (yellow; N=523). **(B)** Histogram indicating the range of nearest distances of equator-facing sections to the equator + poleward facing sections. 479 of 523 (92%) of equator-facing sections occur within 50 km of equator + poleward facing sections, a vast majority of those (66%) occurring within 25 km. The base map is a MOLA shaded relief, illumination from the upper left (256 pixels/degree, credit: MOLA Science Team, MSS, JPL, NASA). We produced this figure using Esri’s ArcGIS 10.3 (<http://www.esri.com/software/arcgis>).


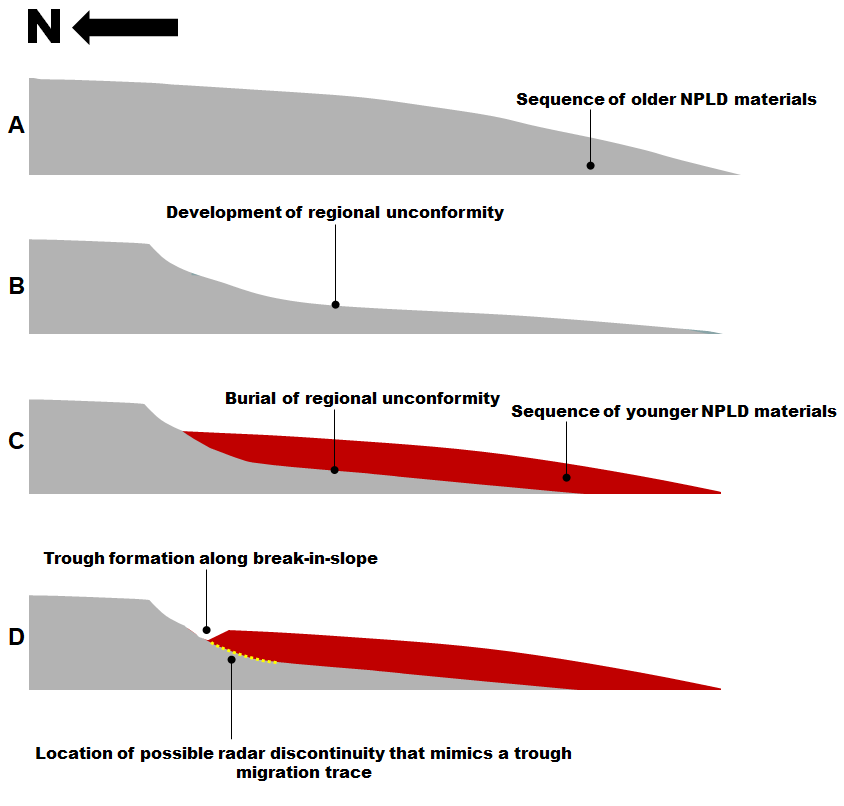
**Figure S13** Sketches illustrating a geologic scenario in which in-situ eroded troughs are developed above older unconformities, which in radar data might, in some cases, resemble trough migration traces.
